# Vitamin D Deficiency Rickets and COVID-19 Pandemic

**DOI:** 10.1155/2021/5512668

**Published:** 2021-04-15

**Authors:** Guwani Liyanage, Yashica de Silva

**Affiliations:** ^1^Department of PaediatricsFaculty of Medical Sciences, University of Sri Jayewardenepura, Nugegoda, Sri Lanka; ^2^Paediatric Professorial Unit, Colombo South Teaching Hospital, Colombo, Sri Lanka

## Abstract

In a breastfed infant, the main source of vitamin D comes from the mother. Thus, maternal vitamin D deficiency is the key reason for vitamin D deficiency (VDD) and rickets during infancy. As they grow older, inadequate sun exposure, diet and lack of supplements also contribute. Individuals with darker skin require at least three to five times longer exposure to the sun than a person with lighter skin to produce adequate endogenous vitamin D. Not many food items naturally contain vitamin D; most of those are less affordable to the poor. We report an 18-month-old child with vitamin D deficiency rickets during strict self-isolation measures during the coronavirus disease 2019 (COVID-19) pandemic. Prolonged periods of confining indoors, low dietary intake of vitamin D, economic distress, maternal deficiency, and nonsupplementation could have contributed to vitamin D deficiency rickets in this child. During an unprecedented pandemic of this nature, simple sun exposure and diet advice may suffice for most. This case report highlights the importance of strengthening individuals and communities with information and formulating strong public health policies to prevent vitamin D deficiency.

## 1. Introduction

Rickets was first described by Glisson et al. in 1650 [1]. Maternal vitamin D deficiency is the key reason for rickets during infancy. As they grow older, inadequate sun exposure and diet and lack of supplements contribute. Individuals with darker skin require at least three to five times longer exposure to the sun than a person with lighter skin to produce adequate endogenous vitamin D [2]. Not many food items naturally contain vitamin D; most of those are less affordable to the poor. We report an 18-month-old child with vitamin D deficiency rickets during strict self-isolation measures during the COVID-19 pandemic.

## 2. Case Report

An 18-month-old male infant with dark skin pigmentation presented with bowlegs in late December 2020. He was from a low-income family and lived in flats in an urban city. Since the child started walking, his mother had concerns about his bowlegs. However, she was late in seeking medical attention due to strict lockdown measures in the country. She was a homemaker, rarely went outdoors, and never consumed vitamin D supplements. The child was born at term and was exclusively breastfed until six months of age. His diet was lacking in nutrients. A 7-day food frequency assessment showed inadequate vitamin D intake. He was not given fish, rarely received fortified food or milk, and was continued to be breastfed. Moreover, the child was confined to the house from mid-March 2020, since the lockdown.

The child's development milestones were normal, and anthropometric parameters were within normal limits (weight, 10.2 kg (median and -1SD); occipitofrontal diameter, 43 cm (-2SD and -3SD); length, 73 cm (-2SD and -3SD)). His length was within the midparental height range. He was alert and playful. Several bone deformities were noted: Harrison sulcus, widened wrists and ankles, and curved legs. His head shape and spine were normal.

The results of laboratory analysis of blood were as follows: serum-corrected calcium, 2.48 mmol/L; phosphorus, 3.2 mg/dL; alkaline phosphatase, 863.9 U/L; 25-OH-vitamin D, 8 ng/mL. The serum parathyroid hormone (PTH) level was high (126.5 pg/mL). The liver enzyme (aspartate transaminase, alanine transaminase, and gamma-glutamyl transpeptidase) levels and renal function test results were within normal limits. The mother's serum 25-OH-vitamin D level was 12.4 ng/mL, and the PTH level was 174.9 pg/mL.

Furthermore, the child showed evidence of iron deficiency. A complete blood count revealed the following: white blood cell count, 12.02 × 10^9^/L, hemoglobin, 11.3 g/dL; mean cell volume, 70.3 fL; mean cell hemoglobin, 23.4 pg; mean cell hemoglobin concentration, 33.3 g/dL; platelet count, 523 × 10^9^/L; and red cell distribution width, 17.1%. A blood picture suggested iron deficiency. He had low iron stores (ferritin, 4.58 ng/mL; iron, 4.4 *µ*mol/L; total iron-binding capacity, 70%; transferrin saturation, 6.28%).

**Figure 1:**
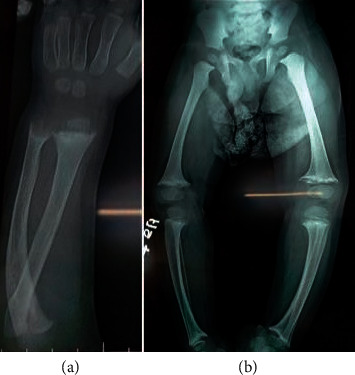
Radiographs showing cupping, fraying, and splaying of the distal radial, ulnar, femoral, and tibial metaphyses.

Radiological evaluation revealed cupping, fraying, and widening of the distal ends of the radii, distal and proximal ends of the tibiae and fibulae, and distal ends of the femurs (Figure 1).

The diagnoses of vitamin D deficiency rickets and iron deficiency were confirmed based on clinical, biochemical, and radiological evidence. The child was administered 2000 IU of cholecalciferol, 500 mg of oral calcium, and 60 mg of iron sulphate. The mother was referred to an endocrinologist and administered vitamin D and calcium therapy.

## 3. Discussion

Rickets is not a disease of the past. Incidence is on the rise, probably owing to lack of exposure to adequate amounts of sunlight with lifestyle changes, particularly in urbanized cities [3]. This case report aims to highlight that the incidence could rise further during this unprecedented COVID-19 pandemic. Multiple factors probably had contributed to rickets in our patient. He was not on supplements and exclusively breastfed for six months. Also, his dietary vitamin D intake was low. His mother was not supplemented during pregnancy or lactation, and she had restricted outdoor activities and low sun exposure. Besides, the child was confined to the house for over nine months with inadequate sun exposure. Hence, it is plausible to think that he had some biochemical deficiency in the early months. It was probably aggravated subsequently by the pandemic events, leading to overt deficiency with clinical rickets.

Children face many primary and secondary consequences of the pandemic. Severe acute respiratory syndrome coronavirus 2 (SARS-CoV-2) mainly affected the respiratory system and many other organs (viz., brain and skin) [4, 5]. The secondary effects of SARS-CoV-2 were disturbance to formal education, child protection issues, domestic violence, and food insecurity. Most importantly, people also faced other health-related adversities, such as macronutrient and micronutrient deficiency [6].

Rickets is a failure of growing bone to mineralize. Diagnosis depends on history, clinical features, radiograph findings, and biochemical analysis [7]. Rickets is manifested with bony deformities and short stature. Craniotabes (softening of bones of the skull) is seen early in infancy. Also, delayed closure of fontanelle and frontal bossing are observed. In older children, rickety rosary, widening of wrists and ankles, bowlegs, or knock knees could be found. Delayed teeth, delayed motor development, hypotonia, and irritability are few nonskeletal features. Radiographs are considered the gold standard for diagnosis.

Vitamin D is produced in the skin upon exposure to ultraviolet radiation (UVB). Then, it will go through 2 stages of hydroxylation before it becomes metabolically active. The skin production of vitamin D depends on many factors such as time of exposure, time of the day, and season [2]. Also, individual variations such as clothing, sunscreen, and age are influential. An adult in a bathing suit, exposed to one minimal erythemal dose of sunlight (sun exposure that produces minimal erythema), produces vitamin D equal to an intake of approximately 20,000 IUs of vitamin D (Hollick's rule). [2]. In urban areas with less outdoor space, exposure of the face and hands over an open window or balcony, between 10 am and 3 pm, would enable endogenous vitamin D production [8, 9]. Also, one should be aware that UVB rays are absorbed by glass and staying behind a glass window is not effective.

A small proportion of vitamin D will be obtained from the diet. Natural sources of vitamin D are primarily small/large fish, fortified dairy products and ready-to-eat cereals, sun-exposed mushrooms, and egg yolk. However, most of these items, except small fish and egg yolk, are less affordable to poor socioeconomic backgrounds. Small fish eaten as a whole (including head, organs, and bones) contains significantly greater amount of calcium (960 mg/100 g), iron (3.3 mg/100 g), and vitamin D3 (3.6 microgram/100 g) than larger fish where only the fillet is eaten [10].

Vitamin D supplementation is not routinely carried out in Sri Lanka despite reports of vitamin D deficiency among various age groups. A single-centre, hospital-based study in the Colombo district had reported vitamin D deficiency (<20 ng/ml) without clinical features of rickets or osteomalacia among pregnant (62.9%) and lactating women (49.5%) and in infants (88.6%) [11]. A recent study conducted in a community setting in the Colombo district among pregnant women in their third trimester (unpublished) showed 62.1% of subclinical deficiency. Also, deficiency had been reported among preschool children (34.1%) by Marasinghe et al. [12]. Subclinical deficiency can progress into overt deficiency during a pandemic of this nature.

## 4. Conclusion

Vitamin D deficiency is a severe public health problem. Increasing public awareness regarding simple measures such as sun exposure and diet would suffice to lessen the burden of vitamin D deficiency in most, particularly during a pandemic of this nature. This highlights the importance of strengthening individuals and communities with information and formulating strong public health policies to prevent vitamin D deficiency. Besides, supplementation of the high-risk groups would be worthwhile, and supplementation guidelines should be tailored to the needs of each country or region.

## Data Availability

The data used to support the case report are included within the article.
